# Supplementary material for: Labor Market Outcomes of People with HIV Pre- and Post-Diagnosis in the Netherlands
Source: Nat Commun. 2026 Jan 28;17:1110. doi: 10.1038/s41467-025-67799-x (PMC12855923; doi:10.1038/s41467-025-67799-x)
Supplement: Supplementary file 1 — Supplementary Information [file 41467_2025_67799_MOESM1_ESM.pdf]

# Labor Market Outcomes of People with HIV Pre- and Post-Diagnosis in the Netherlands
# Supplementary Information

Andrei Tuiu Esmée Zwiers Wendy Janssens Vita Jongen
Ard van Sighem Ferdinand Wit Menno Pradhan Marc van der Valk

Table SI-1: Robustness check results

| | Main results (1) | Replacing with 0/1 (2) | Replacing with last observed (3) | 2010-2015 (4) | Only individuals alive by 2022 (5) | Not-yet diagnosed (6) |
|---|---|---|---|---|---|---|
| *Panel A: Employment* | | | | | | |
| ATT | -0.028*** | -0.041*** | -0.032*** | -0.024*** | -0.028*** | -0.054*** |
| | (0.005) | (0.005) | (0.005) | (0.006) | (0.005) | (0.010) |
| Pre-diagnosis mean | 0.719 | 0.719 | 0.719 | 0.722 | 0.727 | 0.719 |
| Observations | 65,560 | 65,560 | 65,560 | 40,920 | 63,852 | 5,960 |
| *Panel B: Work hours* | | | | | | |
| ATT | -0.035*** | -0.047*** | -0.039*** | -0.031*** | -0.035*** | -0.052*** |
| | (0.005) | (0.005) | (0.005) | (0.005) | (0.005) | (0.009) |
| Pre-diagnosis mean | 0.650 | 0.650 | 0.650 | 0.662 | 0.657 | 0.650 |
| Observations | 61,974 | 61,974 | 61,974 | 38,896 | 60,361 | 5,634 |
| *Panel C: Income* | | | | | | |
| ATT | -3,583.8*** | -4,313.1*** | -3,863.4*** | -3,531.7*** | -3468.5*** | -3,671.5*** |
| | (543.2) | (544.1) | (543.5) | (652.5) | (567.3) | (757.8) |
| Pre-diagnosis mean | 40,135.2 | 40,135.2 | 40,135.2 | 41,924.3 | 40,482.2 | 40,135.2 |
| Observations | 64,416 | 64,416 | 64,416 | 40,326 | 62,734 | 5,856 |
| *Panel D: Disability Insurance* | | | | | | |
| ATT | 0.036*** | 0.056*** | 0.037*** | 0.036*** | 0.035*** | 0.039*** |
| | (0.004) | (0.004) | (0.004) | (0.004) | (0.004) | (0.006) |
| Pre-diagnosis mean | 0.078 | 0.078 | 0.078 | 0.077 | 0.075 | 0.078 |
| Observations | 65,560 | 65,560 | 65,560 | 40,920 | 63,852 | 5,960 |

Notes: This table shows the average treatment effects on the treated (ATT) for our robustness checks, estimated using the Callaway-Sant'Anna estimator and aggregation procedure for staggered difference-in-differences [1]. Standard errors are reported in parentheses. The results are reported for each of our main outcomes (employment, work hours, income and disability insurance receipt) and only for the overall population. Column (1) shows our main results, as seen in column (1) of Table 1. Column (2) shows the results from running the analysis after replacing missing post-diagnosis outcomes with 0. Column (3) shows the results after replacing missing outcomes with the last observed value. Column (4) shows the results after restricting the sample to individuals diagnosed between 2010 and 2015. Column (5) shows the results after restricting the sample to individuals who are still alive by the year 2022 (the end of the panel). Column (6) reports the results from using the not-yet diagnosed control group, rather than the general population (the never-diagnosed). The pre-diagnosis mean shows mean employment, work hours, income and disability insurance receipt for the group of people with HIV, measured in the year before diagnosis. The number of observations is defined as the number of individuals observed in the year prior to diagnosis. Robust and asymptotic standard errors are reported in parentheses. The stars represent the results of a standard two-sided t-test for whether the point estimates are statistically different from zero. Statistical significance is denoted as: * $p < 0.10$, ** $p < 0.05$, *** $p < 0.01$, where p represents the p-value resulting from this test.

Figure SI-1: Permutation robustness check

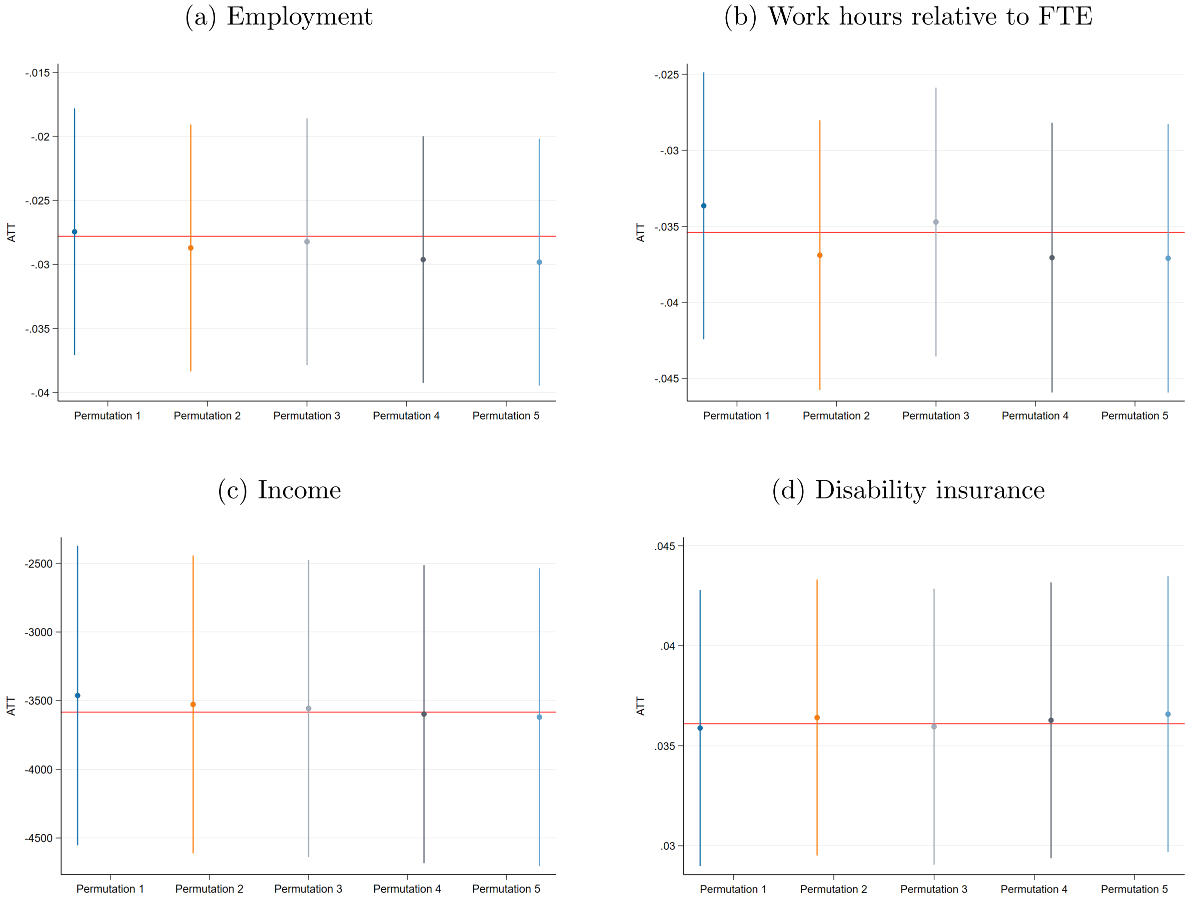


Note: This figure shows the aggregated before-after estimates from running the main analysis with five different randomly drawn samples of controls. The sample of people with HIV is unchanged. Each dot represents an aggregate average treatment effect on the treated estimated using the Callaway-Sant'Anna estimator. Each dot is shown together with 95% confidence intervals. Panel a shows the estimates for employment, panel b for work hours, panel c for income and panel d for disability insurance take-up. In each panel, the red horizontal line shows the main results estimate for the respective outcome, as shown in Table 2.

Figure SI-2: Honest difference-in-differences robustness check

(a) Employment

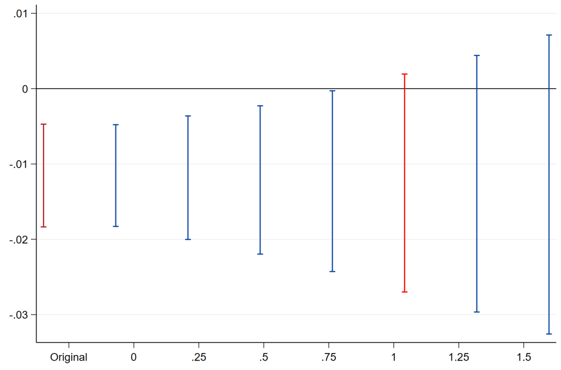


(b) Work hours relative to FTE

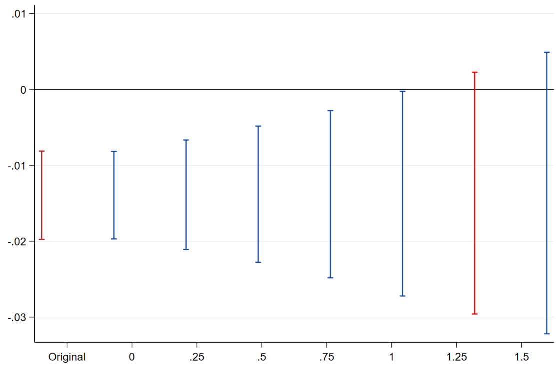


(c) Income

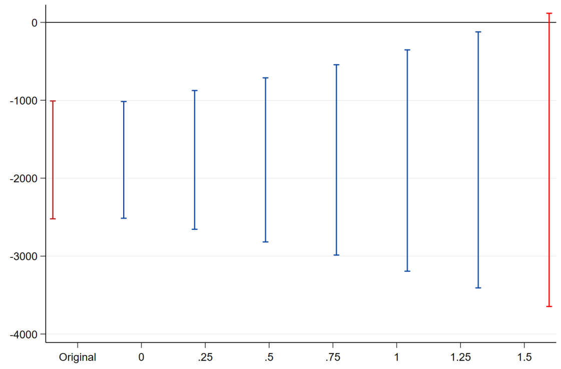


(d) Disability insurance

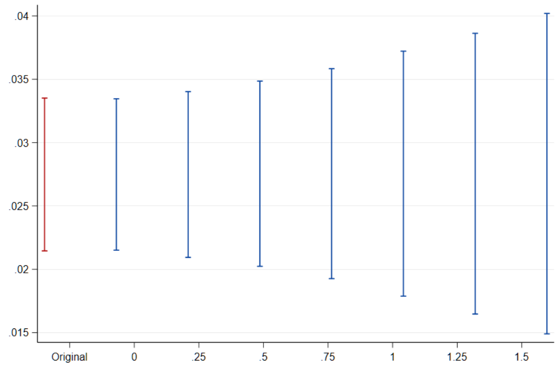


This figure shows the results from running the Honest Difference-in-differences analysis following Rambachan and Roth (2022) on our main results. This allows us to bound our estimated treatment effects while flexibly accounting for potential violations of the parallel trends assumption. Thus, we can identify the "breakdown magnitude" of the violation, the value at which we can no longer reject the null hypothesis that our treatment effect si equal to zero. The X-axis in each figure shows different magnitudes of the violation (relative to the maximum pre-diagnosis drift). We highlight in red the breakdown magnitude.

## Figure SI-3: Employment by age group

(a) Aged below 45 at diagnosis (b) Aged above 45 at diagnosis

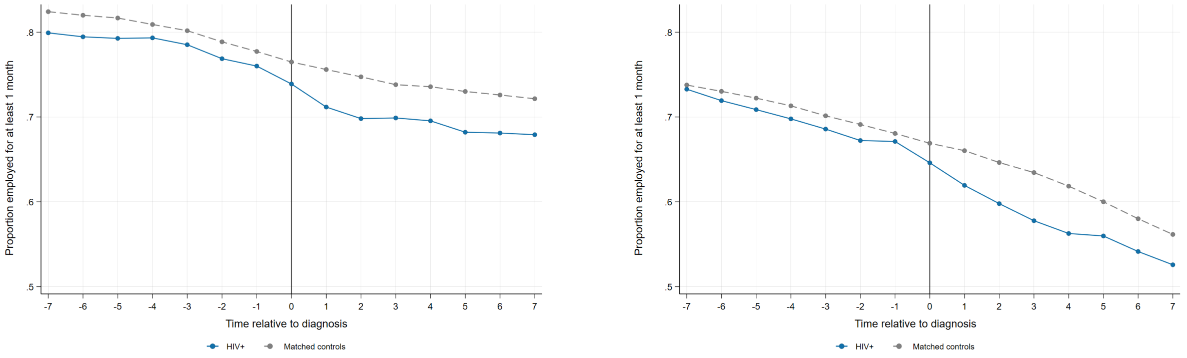


Note: Employment is defined as having income from employment for at least one month in a year. Panel a shows the proportion of employed individuals over time relative to diagnosis (at $t = 0$) for people with HIV and matched controls aged below 45 at diagnosis. Panel b shows the proportion of employed individuals over time relative to diagnosis (at $t = 0$) for people with HIV and matched controls aged above 45 at diagnosis.

## Figure SI-4: Work hours relative to FTE by age group

(a) Aged below 45 at diagnosis (b) Aged above 45 at diagnosis

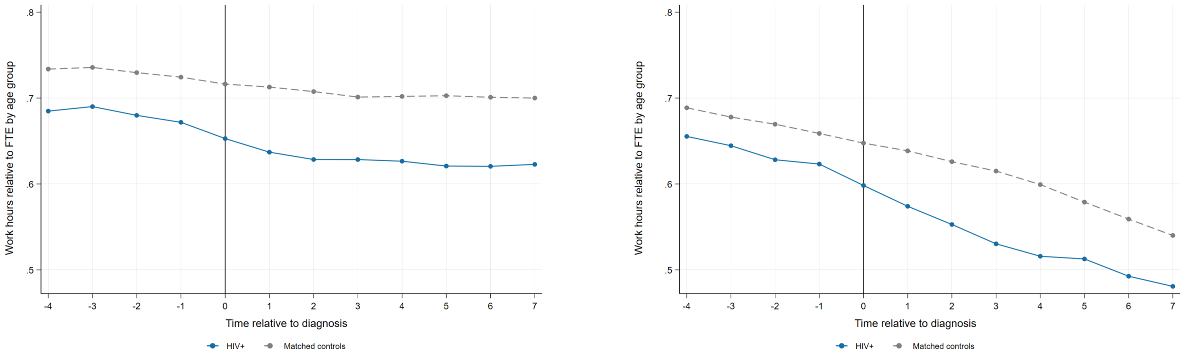


Note: Hours worked is defined in full-time equivalents (FTE), which implies that a value of one represents a full-time job, and zero that the individual does not work. Panel a shows mean work hours relative to FTE, over time relative to diagnosis (at $t = 0$) for people with HIV and matched controls aged below 45 at diagnosis. Panel b shows mean work hours relative to FTE, over time relative to diagnosis (at $t = 0$) for people with HIV and matched controls aged above 45 at diagnosis.

Figure SI-5: Income by age group

(a) Aged below 45 at diagnosis

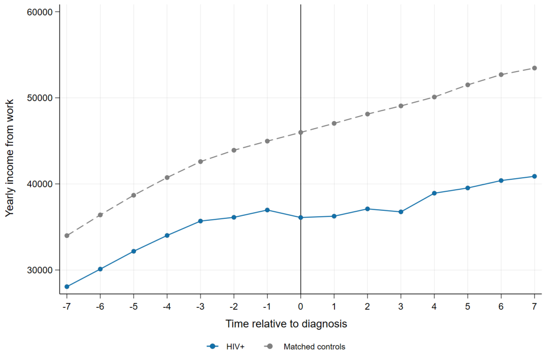


(b) Aged above 45 at diagnosis

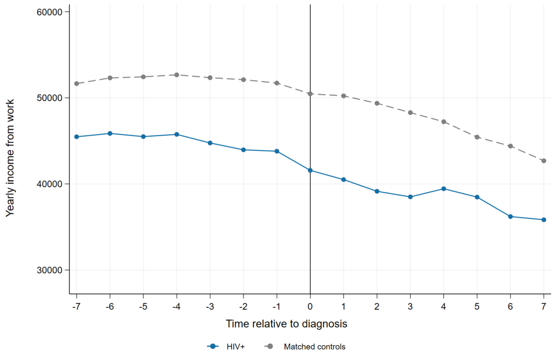


Note: Income is defined as gross annual income from work and self-employment. Panel a shows mean income over time relative to diagnosis (at $t = 0$) for people with HIV and matched controls aged below 45 at diagnosis. Panel b shows mean income over time relative to diagnosis (at $t = 0$) for people with HIV and matched controls aged above 45 at diagnosis.

Figure SI-6: Disability insurance receipt by age group

(a) Aged below 45 at diagnosis

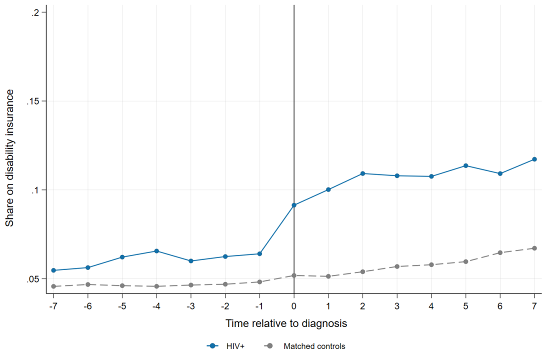


(b) Aged above 45 at diagnosis

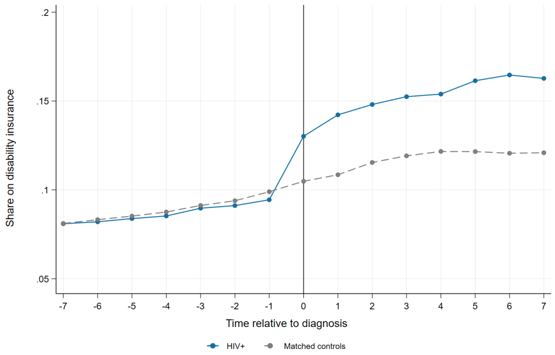


Note: Disability insurance (DI) take-up is defined as receiving disability or sickness benefits for at least one month in a given year. Panel a shows the proportion of individuals receiving DI over time relative to diagnosis (at $t = 0$) for people with HIV and matched controls aged below 45 at diagnosis. Panel b shows the proportion of individuals receiving DI over time relative to diagnosis (at $t = 0$) for people with HIV and matched controls aged above 45 at diagnosis.

Figure SI-7: Employment by migration origin

(a) Native Dutch

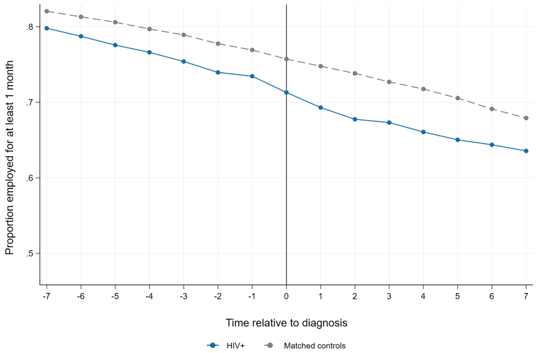


(b) European, North American

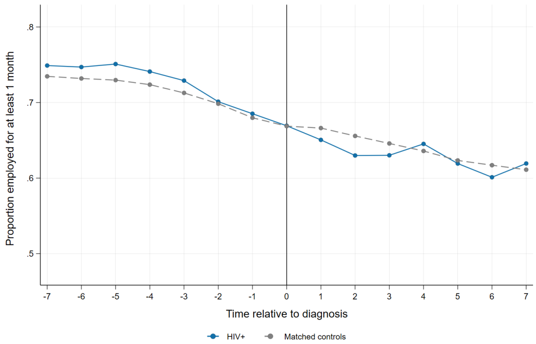


(c) Middle-eastern, North African, Asian

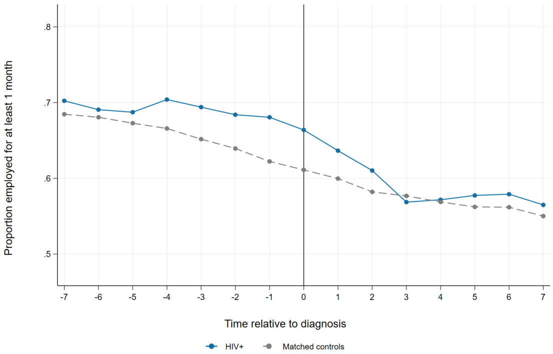


(d) Surinamese, Caribbean, Latin American

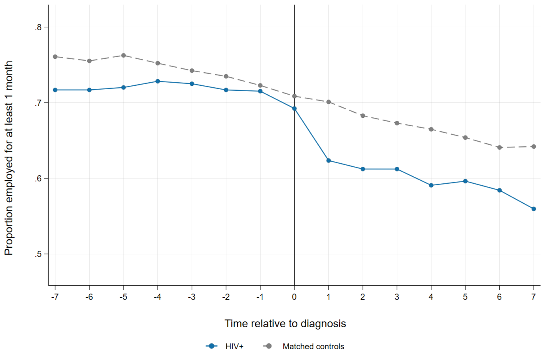


(e) Sub-Saharan African

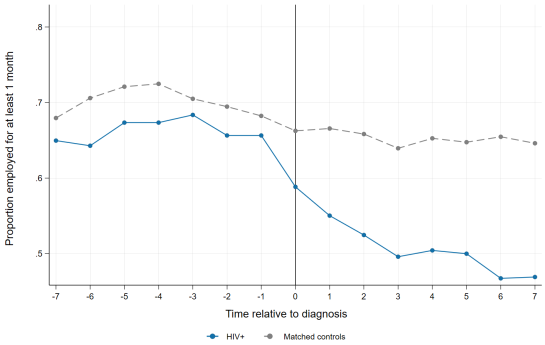


Note: Employment is defined as having income from employment for at least one month in a year. Panel a shows the proportion of employed individuals over time relative to diagnosis (at $t = 0$) for people with HIV and matched controls who are native Dutch (the individual and both parents born in the Netherlands). Panel b shows the proportion of employed individuals over time relative to diagnosis (at $t = 0$) for people with HIV and matched controls with a European or North American migration origin (). Panel c shows the proportion of employed individuals over time relative to diagnosis (at $t = 0$) for people with HIV and matched controls with a Middle-eastern, North African or Asian migration origin (the individual or at least one parent born in one of these regions). Panel d shows the proportion of employed individuals over time relative to diagnosis (at $t = 0$) for people with HIV and matched controls with a Surinamese, Caribbean or Latin American migration origin (the individual or at least one parent born in one of these regions). Panel e shows the proportion of employed individuals over time relative to diagnosis (at $t = 0$) for people with HIV and matched controls with a Sub-Saharan African migration origin (at least one parent born in this region)

Figure SI-8: Work hours relative to FTE by migration origin

(a) Native Dutch

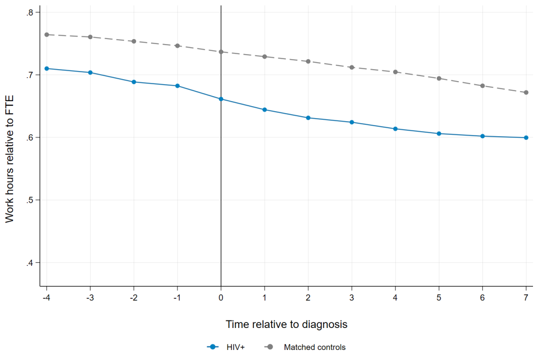


(b) European, North American

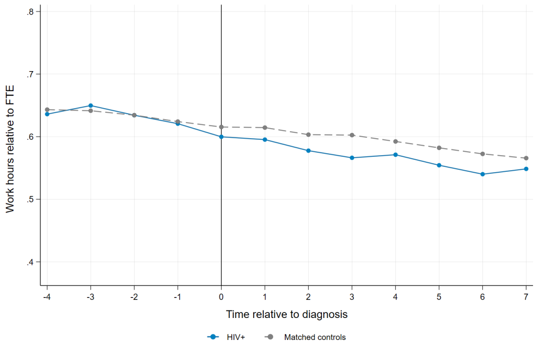


(c) Middle-eastern, North African, Asian

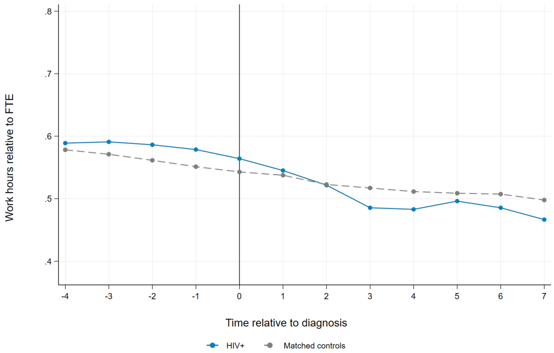


(d) Surinamese, Caribbean, Latin American

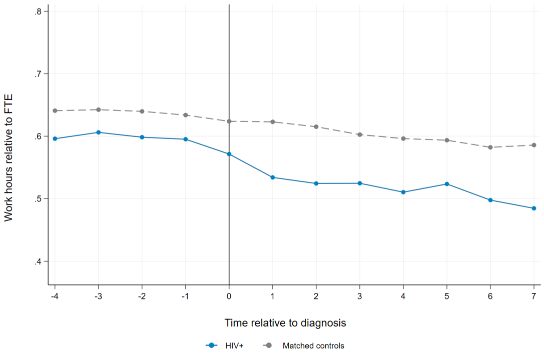


(e) Sub-Saharan African

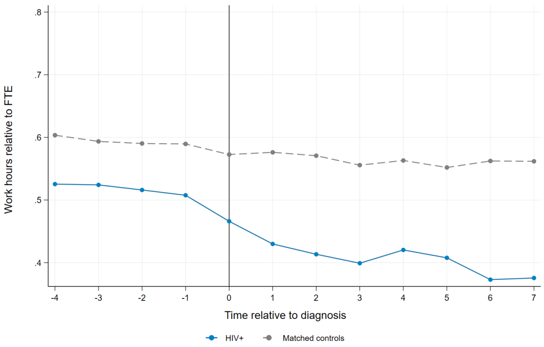


Note: Hours worked is defined in full-time equivalents (FTE), which implies that a value of one represents a full-time job, and zero that the individual does not work. Panel a shows mean work hours relative to FTE, over time relative to diagnosis (at $t = 0$) for people with HIV and matched controls who are native Dutch (the individual and both parents born in the Netherlands). Panel b shows mean work hours relative to FTE, over time relative to diagnosis (at $t = 0$) for people with HIV and matched controls with a European or North American migration origin (the individual or at least one parent born in one of these regions). Panel c shows the proportion of employed individuals over time relative to diagnosis (at $t = 0$) for people with HIV and matched controls with a Middle-eastern, North African or Asian migration origin (the individual or at least one parent born in one of these regions). Panel d shows the proportion of employed individuals over time relative to diagnosis (at $t = 0$) for people with HIV and matched controls with a Surinamese, Caribbean or Latin American migration origin (the individual or at least one parent born in one of these regions). Panel e shows the proportion of employed individuals over time relative to diagnosis (at $t = 0$) for people with HIV and matched controls with a Sub-Saharan African migration origin (at least one parent born in this region)

## Figure SI-9: Income by migration origin

(a) Native Dutch

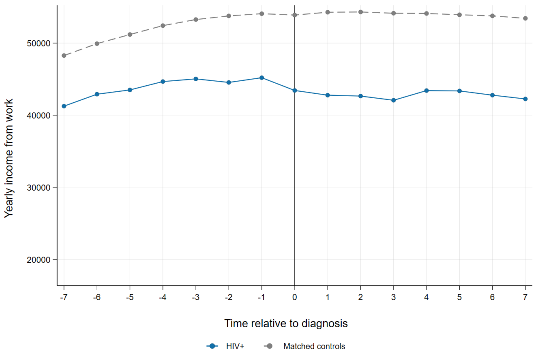


(b) European, North American

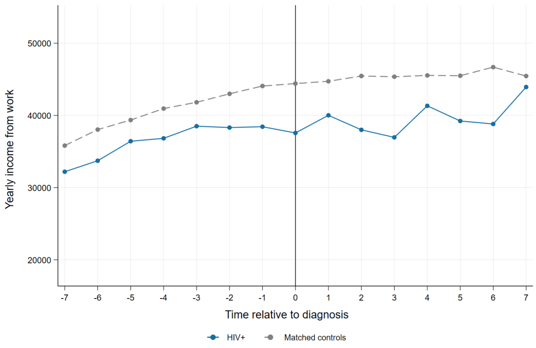


(c) Middle-eastern, North African, Asian

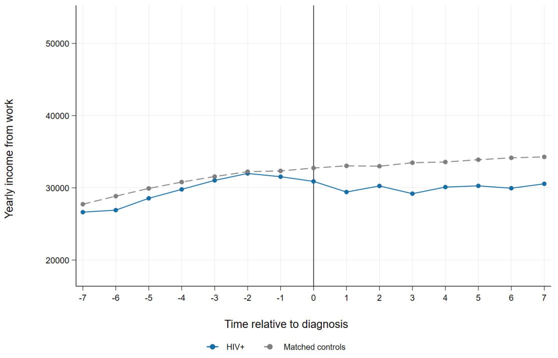


(d) Surinamese, Caribbean, Latin American

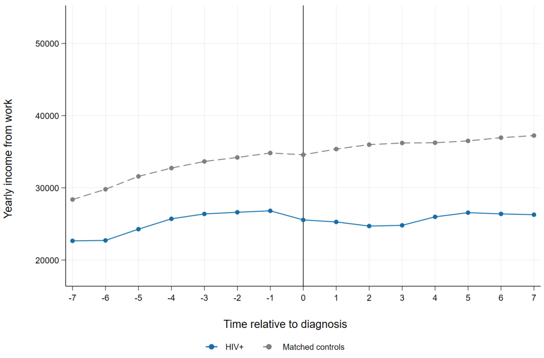


(e) Sub-Saharan African

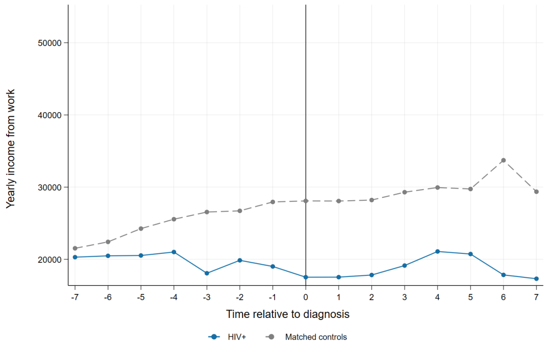


Note: Income is defined as gross annual income from work and self-employment. Panel a shows mean income over time relative to diagnosis (at $t = 0$) for people with HIV and matched controls who are native Dutch (the individual and both parents born in the Netherlands). Panel b shows mean income over time relative to diagnosis (at $t = 0$) for people with HIV and matched controls aged with a European or North American migration origin (the individual or at least one parent born in one of these regions). Panel c shows mean income over time relative to diagnosis (at $t = 0$) for people with HIV and matched controls aged with a Middle-eastern, North African or Asian migration origin (the individual or at least one parent born in one of these regions). Panel d shows mean income over time relative to diagnosis (at $t = 0$) for people with HIV and matched controls aged with a Surinamese, Caribbean or Latin American migration origin (the individual or at least one parent born in one of these regions). Panel e shows mean income over time relative to diagnosis (at $t = 0$) for people with HIV and matched controls aged with a Sub-Saharan African migration origin (at least one parent born in this region)

Figure SI-10: Disability insurance receipt by migration origin

(a) Native Dutch

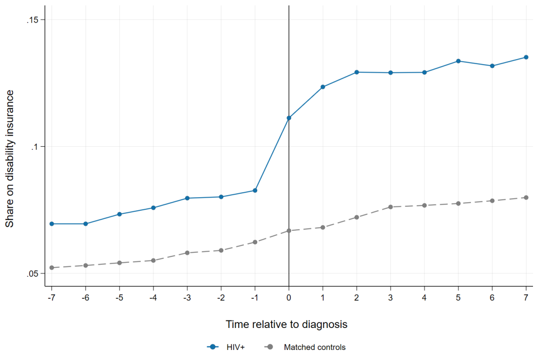


(b) European, North American

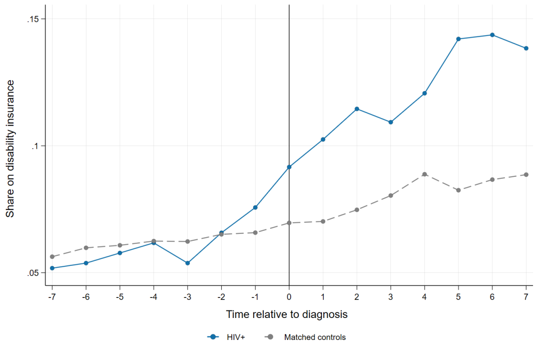


(c) Middle-eastern, North African, Asian

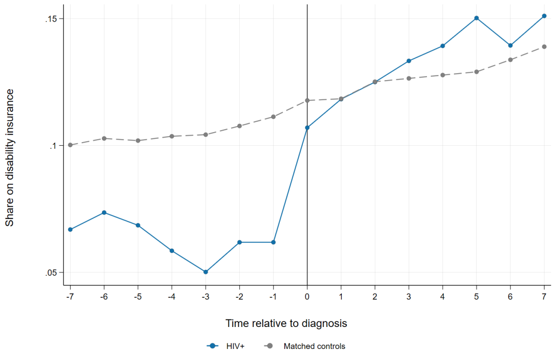


(d) Surinamese, Caribbean, Latin American

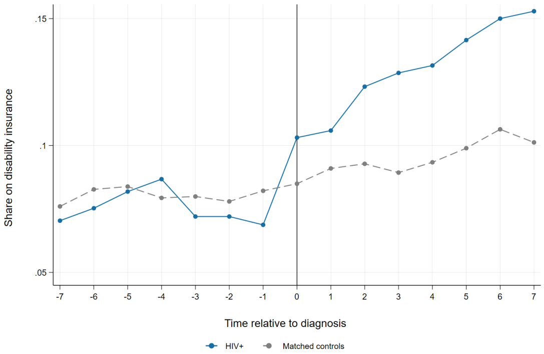


(e) Sub-Saharan African

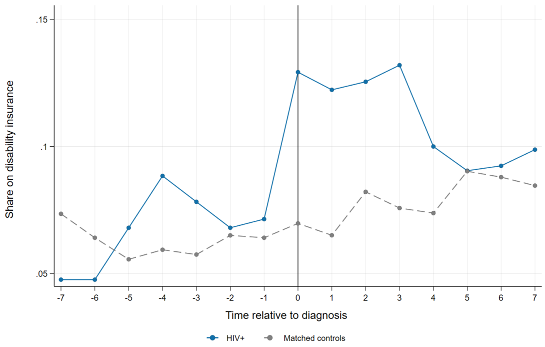


Note: Disability insurance (DI) take-up is defined as receiving disability or sickness benefits for at least one month in a given year. Panel a shows the proportion of individuals receiving DI over time relative to diagnosis (at $t = 0$) for people with HIV and matched controls who are native Dutch (the individual and both parents born in the Netherlands). Panel b shows the proportion of individuals receiving DI over time relative to diagnosis (at $t = 0$) for people with HIV and matched controls aged with a European or North American migration origin (the individual or at least one parent born in one of these regions). Panel c shows the proportion of individuals receiving DI over time relative to diagnosis (at $t = 0$) for people with HIV and matched controls aged with a Middle-eastern, North African or Asian migration origin (the individual or at least one parent born in one of these regions). Panel d shows the proportion of individuals receiving DI over time relative to diagnosis (at $t = 0$) for people with HIV and matched controls aged with a Surinamese, Caribbean or Latin American migration origin (the individual or at least one parent born in one of these regions). Panel e shows the proportion of individuals receiving DI over time relative to diagnosis (at $t = 0$) for people with HIV and matched controls aged with a Sub-Saharan African migration origin (at least one parent born in this region)

Figure SI-11: Employment by stage at diagnosis

(a) Recent/early

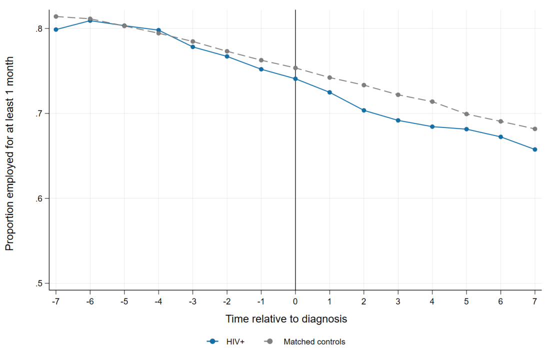


(b) CD4 cell count above 350

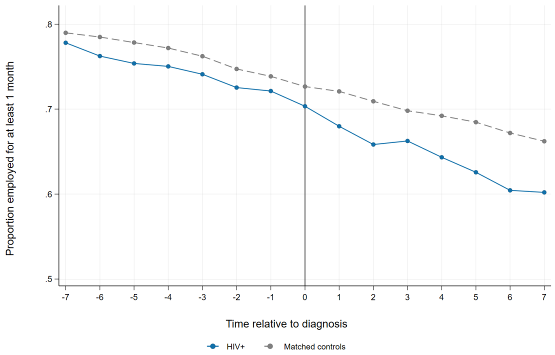


(c) CD4 cell count between 200 and 350

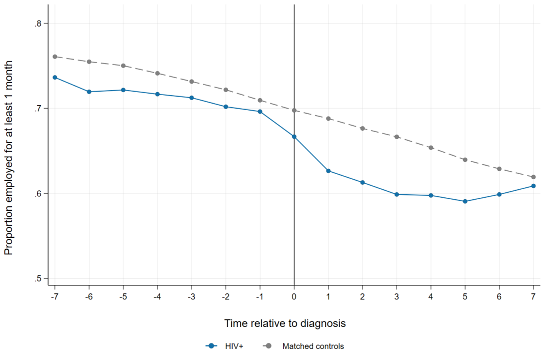


(d) CD4 cell count below 200

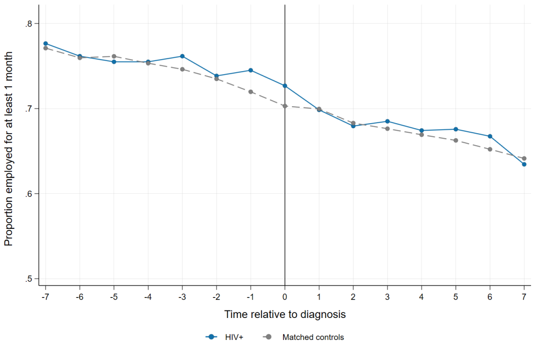


(e) AIDS at diagnosis

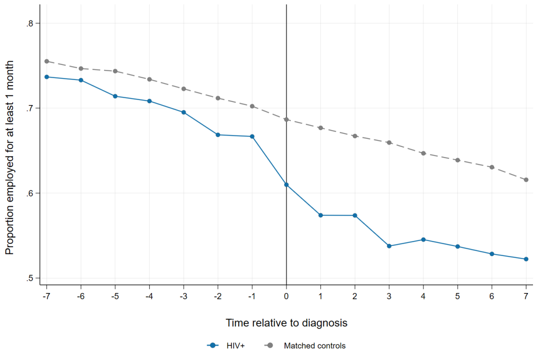


Note: Employment is defined as having income from employment for at least one month in a year. Panel a shows the proportion of employed individuals over time relative to diagnosis (at $t = 0$) for people with HIV a recent infection, as defined by having had a negative HIV test within 12 months prior to diagnosis, and their matched controls. Panel b shows the proportion of employed individuals over time relative to diagnosis (at $t = 0$) for people with HIV with a CD4 cell count above 350 cells/mm$^3$ of blood, and their matched controls. Panel c shows the proportion of employed individuals over time relative to diagnosis (at $t = 0$) for people with HIV with a CD4 cell count between 200 and 350 cells/mm$^3$ of blood, and their matched controls. Panel d shows the proportion of employed individuals over time relative to diagnosis (at $t = 0$) for people with HIV with a CD4 cell count below 200 cells/mm$^3$ of blood, and their matched controls. Panel e shows the proportion of employed individuals over time relative to diagnosis (at $t = 0$) for people with HIV with AIDS-defining illnesses at diagnosis, and their matched controls.

Figure SI-12: Work hours relative to FTE by stage at diagnosis

(a) Recent/early

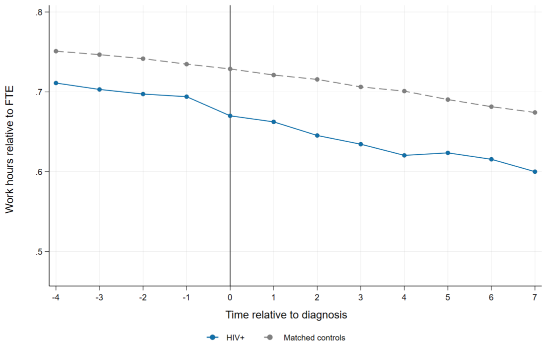


(b) CD4 cell count above 350

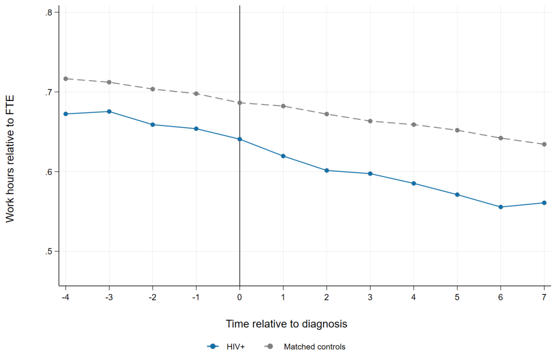


(c) CD4 cell count between 200 and 350

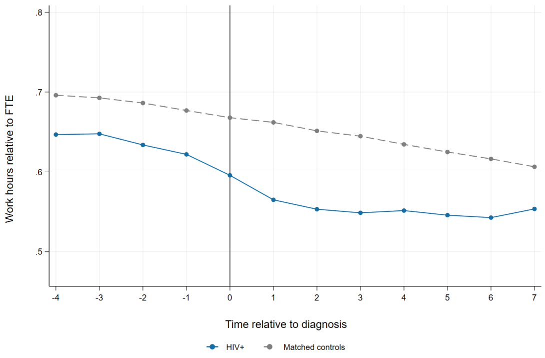


(d) CD4 cell count below 200

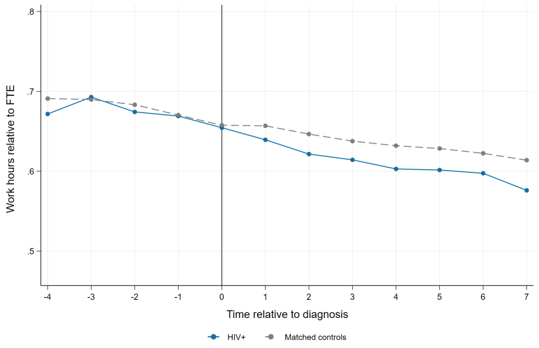


(e) AIDS at diagnosis

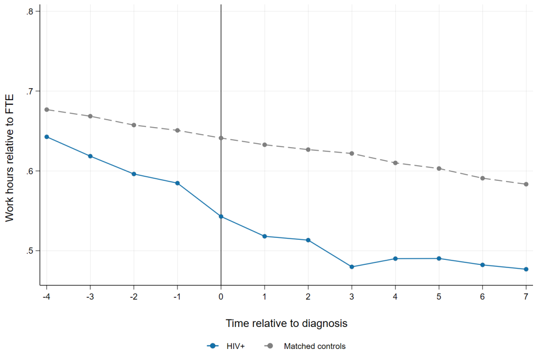


Note: Hours worked is defined in full-time equivalents (FTE), which implies that a value of one represents a full-time job, and zero that the individual does not work. Panel a shows mean work hours relative to FTE, over time relative to diagnosis (at $t = 0$) for people with HIV a recent infection, as defined by having had a negative HIV test within 12 months prior to diagnosis, and their matched controls. Panel b shows mean work hours relative to FTE, over time relative to diagnosis (at $t = 0$) for people with HIV with a CD4 cell count above 350 cells/mm$^3$ of blood, and their matched controls. Panel c shows mean work hours relative to FTE, over time relative to diagnosis (at $t = 0$) for people with HIV with a CD4 cell count between 200 and 350 cells/mm$^3$ of blood, and their matched controls. Panel d shows mean work hours relative to FTE, over time relative to diagnosis (at $t = 0$) for people with HIV with a CD4 cell count below 200 cells/mm$^3$ of blood, and their matched controls. Panel e shows mean work hours relative to FTE, over time relative to diagnosis (at $t = 0$) for people with HIV with AIDS-defining illnesses at diagnosis, and their matched controls.

## Figure SI-13: Income by stage at diagnosis

(a) Recent/early

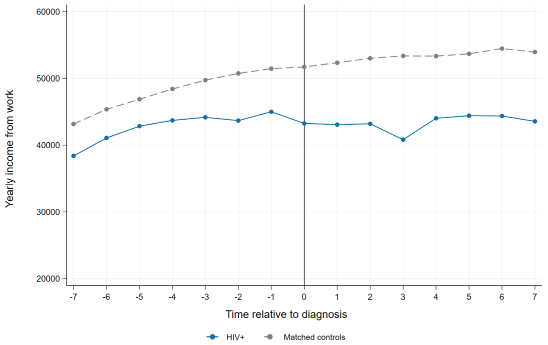


(b) CD4 cell count above 350

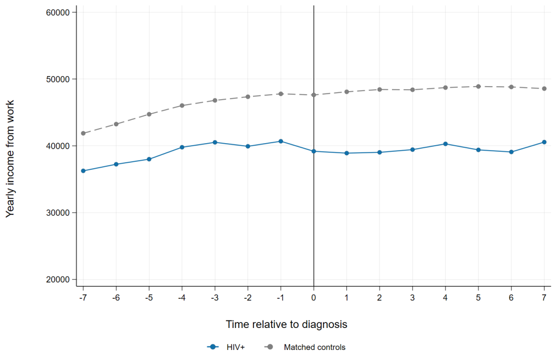


(c) CD4 cell count between 200 and 350

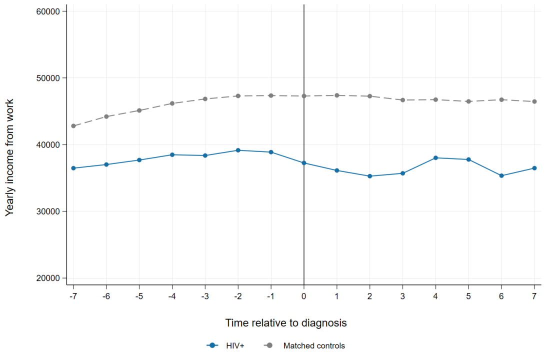


(d) CD4 cell count below 200

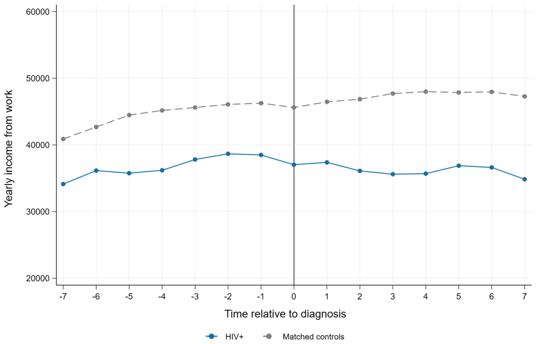


(e) AIDS at diagnosis

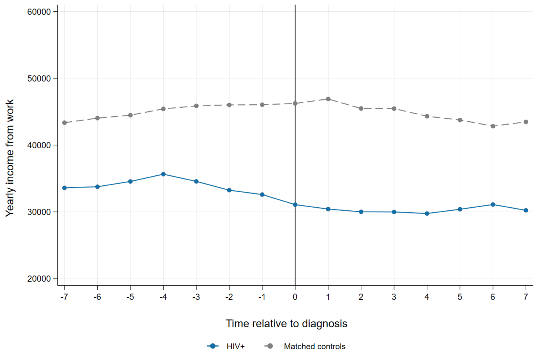


Note: Income is defined as gross annual income from work and self-employment. Panel a shows mean income over time relative to diagnosis (at $t = 0$) for people with HIV a recent infection, as defined by having had a negative HIV test within 12 months prior to diagnosis, and their matched controls. Panel b shows mean income over time relative to diagnosis (at $t = 0$) for people with HIV with a CD4 cell count above 350 cells/mm$^3$ of blood, and their matched controls. Panel c shows mean income over time relative to diagnosis (at $t = 0$) for people with HIV with a CD4 cell count between 200 and 350 cells/mm$^3$ of blood, and their matched controls. Panel d shows mean income over time relative to diagnosis (at $t = 0$) for people with HIV with a CD4 cell count below 200 cells/mm$^3$ of blood, and their matched controls. Panel e shows mean income over time relative to diagnosis (at $t = 0$) for people with HIV with AIDS-defining illnesses at diagnosis, and their matched controls.

Figure SI-14: Disability insurance receipt by stage at diagnosis

(a) Recent/early

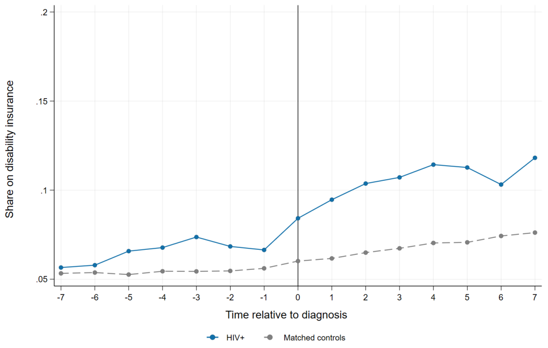


(b) CD4 cell count above 350

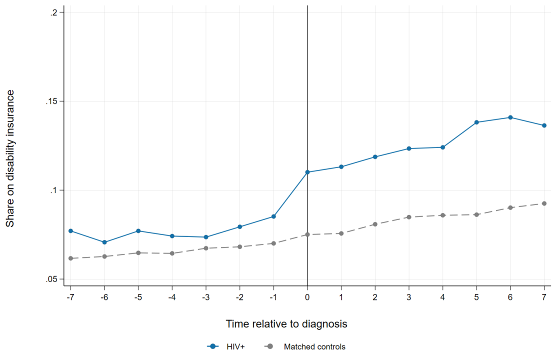


(c) CD4 cell count between 200 and 350

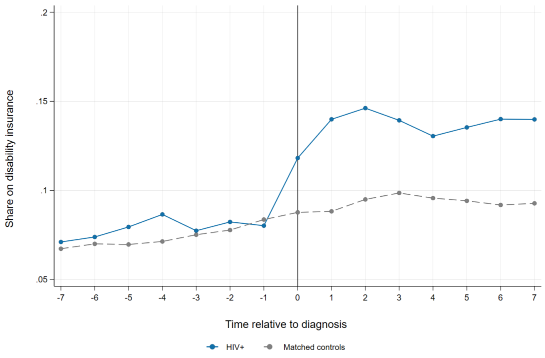


(d) CD4 cell count below 200

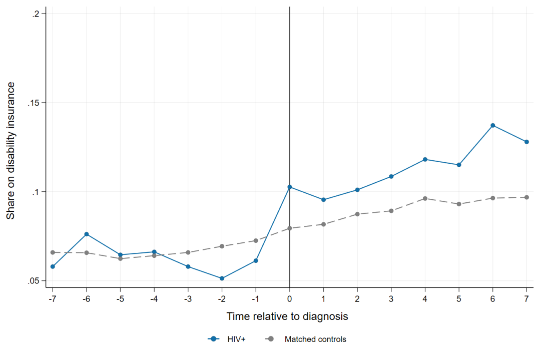


(e) AIDS at diagnosis

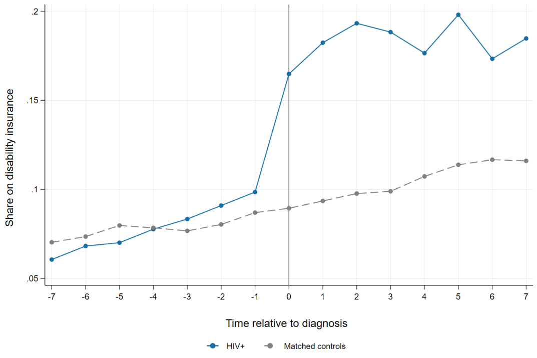


Note: Disability insurance (DI) take-up is defined as receiving disability or sickness benefits for at least one month in a given year. Panel a shows the proportion of individuals receiving DI over time relative to diagnosis (at $t = 0$) for people with HIV a recent infection, as defined by having had a negative HIV test within 12 months prior to diagnosis, and their matched controls. Panel b shows the proportion of individuals receiving DI over time relative to diagnosis (at $t = 0$) for people with HIV with a CD4 cell count above 350 cells/mm$^3$ of blood, and their matched controls. Panel c shows the proportion of individuals receiving DI over time relative to diagnosis (at $t = 0$) for people with HIV with a CD4 cell count between 200 and 350 cells/mm$^3$ of blood, and their matched controls. Panel d shows the proportion of individuals receiving DI over time relative to diagnosis (at $t = 0$) for people with HIV with a CD4 cell count below 200 cells/mm$^3$ of blood, and their matched controls. Panel e shows the proportion of individuals receiving DI over time relative to diagnosis (at $t = 0$) for people with HIV with AIDS-defining illnesses at diagnosis, and their matched controls.

Figure SI-15: Employment by period

(a) Diagnosed before 2015

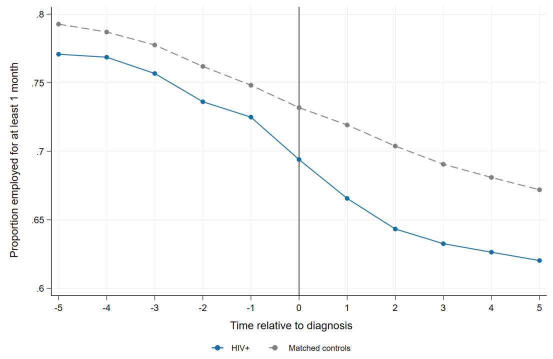


(b) Diagnosed after 2015

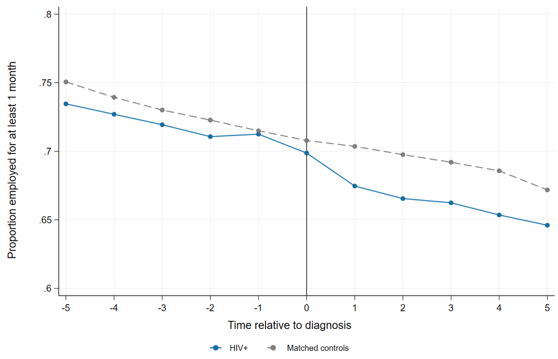


Note: Employment is defined as having income from employment for at least one month in a year. Panel (a) shows the proportion of individuals in employment for at least 1 month over time relative to diagnosis (at t = 0), for people diagnosed before the year 2015 and their matched controls. Panel (b) shows the same outcome for individuals diagnosed in the year 2015 or later. Matched controls are assigned to the same year of diagnosis as their respective matched person with HIV.

Figure SI-16: Work hours by period

(a) Diagnosed before 2015

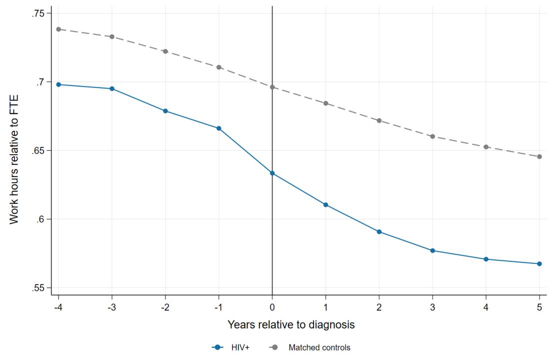


(b) Diagnosed after 2015

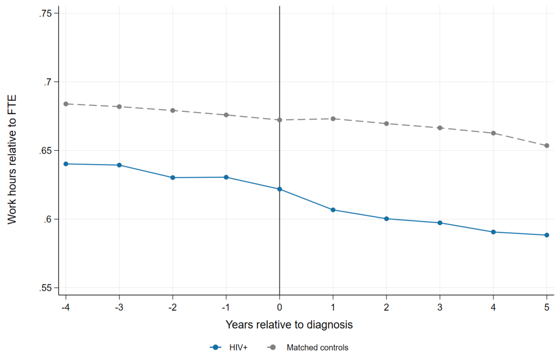


Note: Hours worked is defined in full-time equivalents (FTE), which implies that a value of one represents a full-time job, and zero that the individual does not work. Panel (a) shows the work hours relative to FTE over time relative to diagnosis (at t = 0), for people diagnosed before the year 2015 and their matched controls. Panel (b) shows the same outcome for individuals diagnosed in the year 2015 or later. Matched controls are assigned to the same year of diagnosis as their respective matched person with HIV.

Figure SI-17: Income by period

(a) Diagnosed before 2015 (b) Diagnosed after 2015

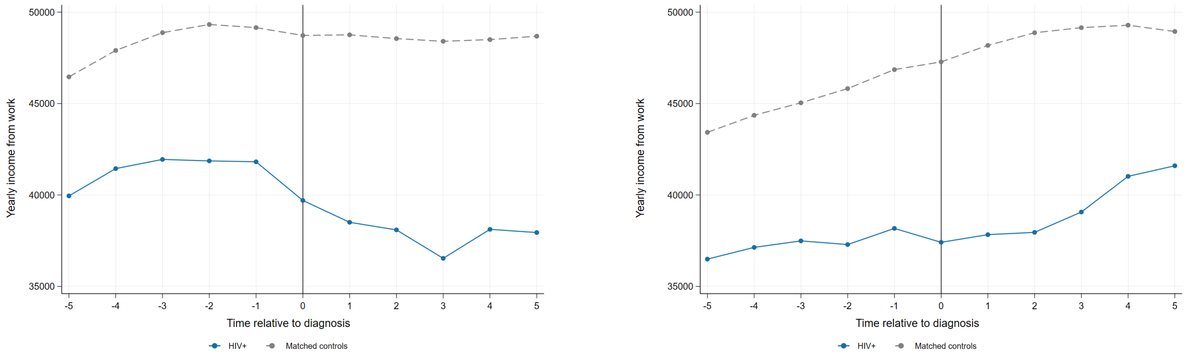


Note: Income is defined as gross annual income from work and self-employment. Panel (a) shows the yearly income from work (in 2015 euros) over time relative to diagnosis (at t = 0), for people diagnosed before the year 2015 and their matched controls. Panel (b) shows the same outcome for individuals diagnosed in the year 2015 or later. Matched controls are assigned to the same year of diagnosis as their respective matched person with HIV.

Figure SI-18: Disability insurance receipt by period

(a) Diagnosed before 2015 (b) Diagnosed after 2015

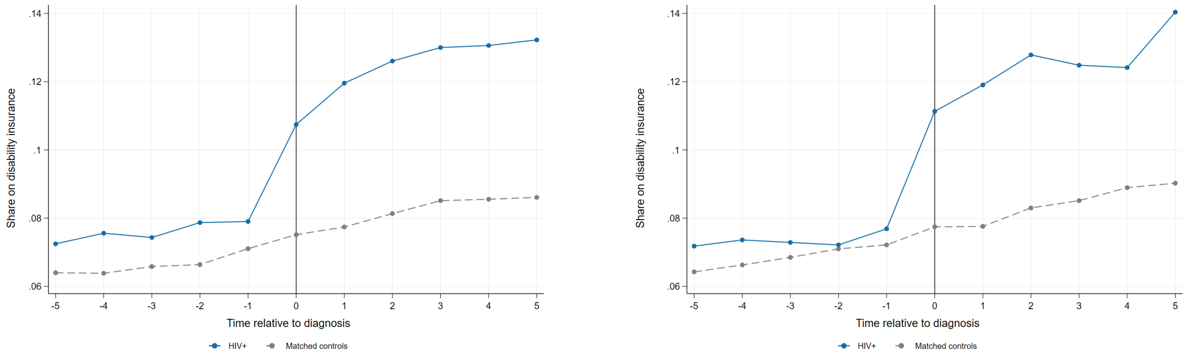


Note: Disability insurance (DI) take-up is defined as receiving disability or sickness benefits for at least one month in a given year. Panel (a) shows the proportion of individuals receiving DI or sickness benefits for at least 1 month over time relative to diagnosis (at t = 0), for people diagnosed before the year 2015 and their matched controls. Panel (b) shows the same outcome for individuals diagnosed in the year 2015 or later. Matched controls are assigned to the same year of diagnosis as their respective matched person with HIV.

Figure SI-19: Employment by gender

(a) Male

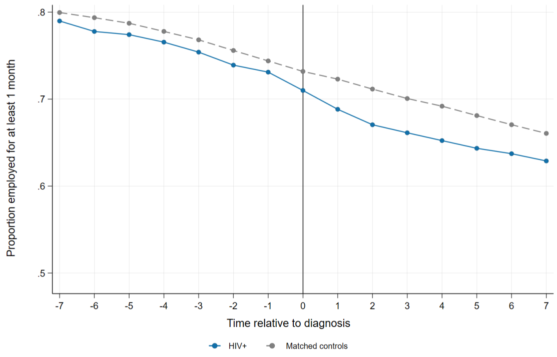


(b) Female

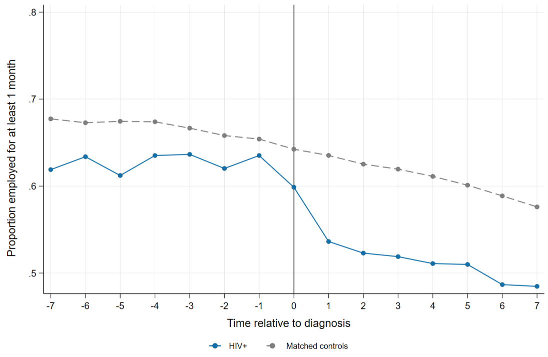


Note: Employment is defined as having income from employment for at least one month in a year. Panel (a) shows the proportion of individuals in employment for at least 1 month over time relative to diagnosis (at t = 0), for individuals registered as males by Statistics Netherlands. Panel (b) shows the same outcome for individuals registered as females. Matched controls are assigned to the same year of diagnosis as their respective matched person with HIV.

Figure SI-20: Work hours by gender

(a) Male

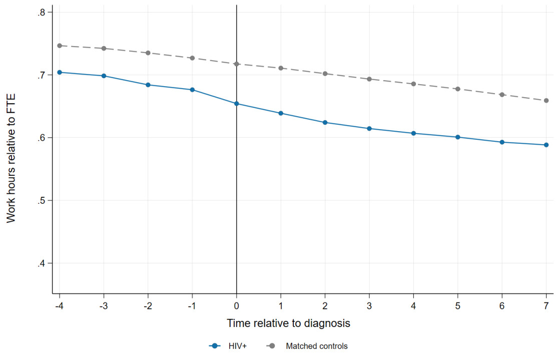


(b) Female

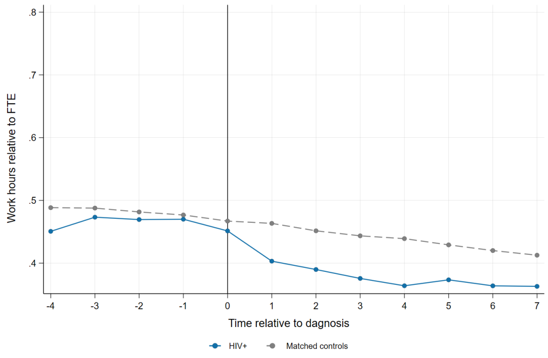


Note: Hours worked is defined in full-time equivalents (FTE), which implies that a value of one represents a full-time job, and zero that the individual does not work. Panel (a) shows the work hours relative to FTE over time relative to diagnosis (at t = 0), for individuals registered as males by Statistics Netherlands. Panel (b) shows the same outcome for individuals registered as females. Matched controls are assigned to the same year of diagnosis as their respective matched person with HIV.

Figure SI-21: Income by gender

(a) Male

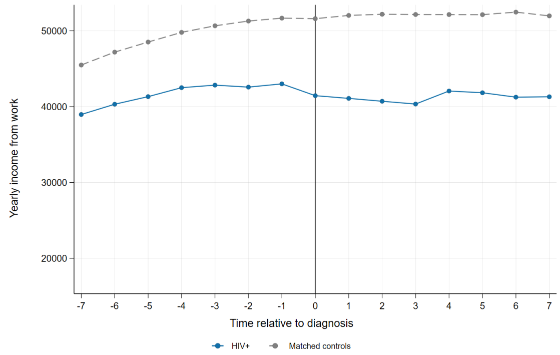


(b) Female

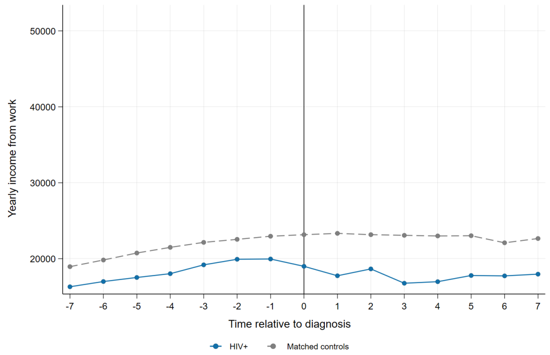


Note: Income is defined as gross annual income from work and self-employment. Panel (a) shows the yearly income from work (in 2015 euros) over time relative to diagnosis (at t = 0), for individuals registered as males by Statistics Netherlands. Panel (b) shows the same outcome for individuals registered as females. Matched controls are assigned to the same year of diagnosis as their respective matched person with HIV.

Figure SI-22: Disability insurance receipt by gender

(a) Male

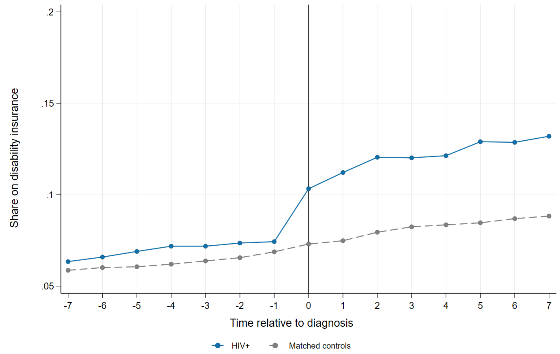


(b) Female

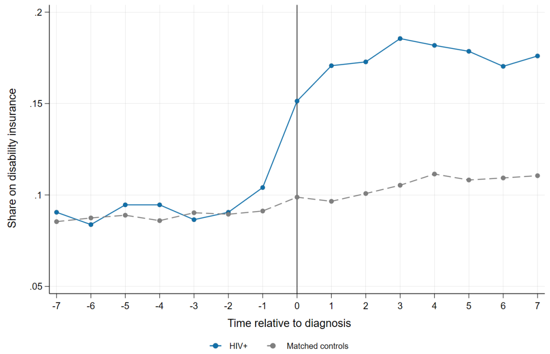


Note: Disability insurance (DI) take-up is defined as receiving disability or sickness benefits for at least one month in a given year. Panel (a) shows the proportion of individuals receiving DI or sickness benefits for at least 1 month over time relative to diagnosis (at t = 0), for individuals registered as males by Statistics Netherlands. Panel (b) shows the same outcome for individuals registered as females. Matched controls are assigned to the same year of diagnosis as their respective matched person with HIV.

Figure SI-23: Mental healthcare costs - extensive and intensive margins

(a) People with HIV and matched controls

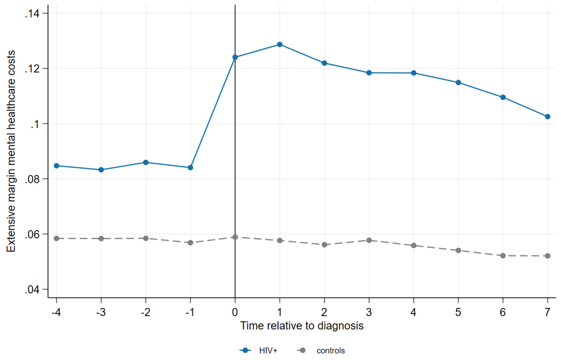


(b) People with HIV and controls by disease stage

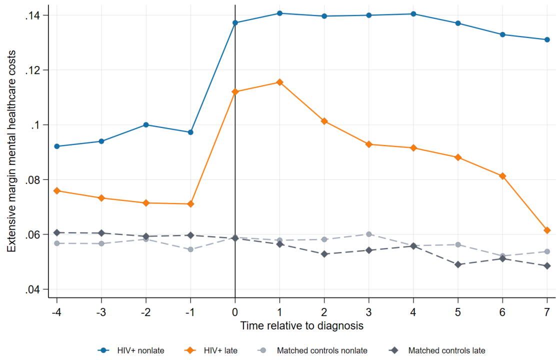


(c) People with HIV and controls

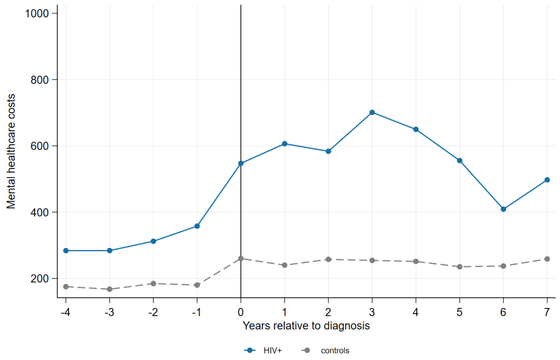


(d) People with HIV and controls disease stage

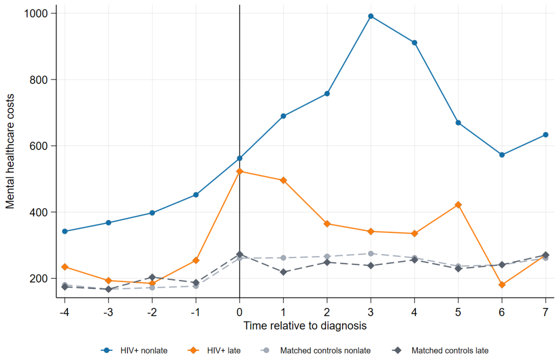


Note: The figure shows mental healthcare costs, as reimbursed through the Dutch basic insurance scheme. These include the costs of diagnosis-treatment combinations for mental healthcare, with or without a hospital stay, which cover mental health issues ranging from mild to severe. It is important to note that the variable only covers the period 2009 – 2022. For this reason, we focus on cohorts diagnosed in the period 2013 - 2022, such that they can be observed for at least four years before diagnosis, and report values from four years before diagnosis to 7 years after diagnosis. In the period 2009 – 2013, mental healthcare costs are contained in a single measure by Statistics Netherlands. However, the variable definition changed in 2014 as mental healthcare costs were split into basic and specialized care. We add up the costs in basic and specialized care between 2014—2022 to compare them to the costs in 2009—2013. Panel a shows the share of individuals with any positive amount of mental health care expenditures (i.e., the extensive margin) over time relative to diagnosis (at $t = 0$) for people with HIV (solid blue line) and matched controls (dashed gray line). Panel b makes an additional distinction between people with HIV and matched controls depending on the stage of HIV at diagnosis: non-late (solid blue line and dashed light gray line, respectively) and late (solid orange line and dark gray dashed line, respectively. Panels c and d show mean expenditures in euros per year. This is shown after removing from the sample individuals whose care expenditures are above the 97.5th percentile of consumers (excluding individuals who do not consume any mental healthcare) at any point in the four years prior to diagnosis. This removes from the sample 30 people with HIV (0.8% of the 3901 people with HIV with non-missing mental healthcare costs at t-1) and 153 controls (0.4% of the 38,801 with non-missing expenditures at t-1). Panel c shows this for all people with HIV and matched controls. Panel d separates the sample by HIV stage at diagnosis, as described above. The small increase observed at the time of diagnosis for the matched controls is caused by the fact only outliers in the pre-diagnosis period are removed from the sample.

## Supplementary Note 1 - ATHENA National Observational HIV Cohort

**Clinical centres**
* denotes site coordinating physician

**Amsterdam UMC, Amsterdam:**
*HIV treating physicians:* F.J.B. Nellen*, M.A. van Agtmael, M. Bomers, G.J. de Bree, S.E. Geerlings, A. Goorhuis, V.C. Harris, J.W. Hovius, B. Lemkes, E.J.G. Peters, T. van der Poll, J.M. Prins, K.C.E. Sigaloff, V. Spoorenberg, M. van der Valk, M. van Vugt, W.J. Wiersinga, F.W.M.N. Wit. *HIV nurse consultants:* C. Bruins, J. van Eden, I.J. Hylkema-van den Bout, L.M. Laan, F.J.J. Pijnappel, S.Y. Smalhout, M.E. Spelbrink, A.M. Weijsenfeld. *HIV clinical virologists/chemists:* N.K.T. Back, R. van Houdt, M. Jonges, S. Jurriaans, F. van someren Gréve, M.R.A. Welkers, K.C. Wolthers.

**Emma Kinderziekenhuis (Amsterdam UMC), Amsterdam:**
*HIV treating physicians:* M. van der Kuip, D. Pajkrt. *HIV nurse consultants:* F.M. Hessing, A.M. Weijsenfeld.

**Admiraal De Ruyter Ziekenhuis, Goes:**
*HIV treating physicians:* M. van den Berge*, A. Stegeman. *HIV nurse consultants:* S. Baas, L. Hage de Looff. *HIV clinical virologists/chemists:* A. van Arkel, J. Stohr, B. Wintermans.

**Catharina Ziekenhuis, Eindhoven:**
*HIV treating physicians:* M.J.H. Pronk*, H.S.M. Ammerlaan.
*HIV nurse consultants:* E.S. de Munnik, S. Phaf.
*HIV clinical virologists/chemists:* B. Deiman, V. Scharnhorst, M.C.A. Wegdam.

**DC Klinieken Lairesse – Hiv Focus Centrum, Amsterdam:**
*HIV treating physicians:* J. Nellen*, A. van Eeden, E. Hoornenborg, S de Stoppelaar. *HIV nurse consultants:* H. Berends, L.J.M. Elsenburg, H. Nobel. *HIV clinical virologists/chemists:* F. van Someren Gréve, M. Welkers, K. Wolthers, N. Back, S. Jurriaans

**ETZ (Elisabeth-TweeSteden Ziekenhuis), Tilburg:**
*HIV treating physicians:* M.E.E. van Kasteren*, M.A.H. Berrevoets, A.E. Brouwer. HIV nurse specialist: A. Adams, B.A.F.M. de Kruijf-van de Wiel. *HIV nurse consultants:* M. Pauwels-van Rijkevoorsel. HIV data collection: B.A.F.M. de Kruijf-van de Wiel. *HIV clinical virologists/chemists:* J.L. Murck.

**Erasmus MC, Rotterdam:**
*HIV treating physicians:* C. Rokx*, A.A. Anas, H.I. Bax, E.C.M. van Gorp, M. de Mendonça Melo, E. van Nood, J.L. Nouwen, B.J.A. Rijnders, C.A.M. Schurink, L. Slobbe, T.E.M.S. de Vries-Sluijs. *HIV nurse consultants:* N. Bassant, J.E.A. van Beek, M. Vriesde, L.M. van Zonneveld. HIV data collection: J. de Groot. *HIV clinical virologists/chemists:* J.J.A. van Kampen, M.P.G Koopmans.

**Erasmus MC Sophia Kinderziekenhuis, Rotterdam:**
*HIV treating physicians:* P.L.A. Fraaij, A.M.C. van Rossum, C.L. Vermont. *HIV nurse consultants:* L.C. van der Knaap.

**Flevoziekenhuis, Almere:**

*HIV treating physicians:* J. Branger*, R.A. Douma. *HIV nurse consultant:* A.S. Cents-Bosma, M.A. Mulder.

**HagaZiekenhuis, Den Haag:**
*HIV treating physicians:* E.F. Schippers*, C. de Bree, C. van Nieuwkoop. *HIV nurse consultants:* J. Geilings, A. van Overeem. HIV data collection: G. van der Hut. *HIV clinical virologists/chemists:* N.D. van Burgel.

**HMC (Haaglanden Medisch Centrum), Den Haag:**
*HIV treating physicians:* E.M.S. Leyten*, L.B.S. Gelinck, F. Mollema. *HIV nurse consultants:* M. Langbein, G.S. Wildenbeest. *HIV clinical virologists/chemists:* T. Nguyen.

**Isala, Zwolle:**
HIV treating physicians: B. Hafkamp*, J.W. Bouwhuis, A.J.J. Lammers. *HIV nurse consultants:* A.G.W. van Hulzen, S. Kraan. *HIV clinical virologists/chemists:* S.B. Debast, G.H.J. Wagenvoort.

**Leids Universitair Medisch Centrum, Leiden:**
*HIV treating physicians:* A.H.E. Roukens*, M.G.J. de Boer, H. Jolink, M.M.C. Lambregts, H. Scheper. *HIV nurse consultants:* A. Metselaar, D. van der Sluis. *HIV clinical virologists/chemists:* S.A. Boers, E.C.J. Claas, E. Wessels.

**Maasstad Ziekenhuis, Rotterdam:**
*HIV treating physicians:* J.G. den Hollander*, R. El Moussaoui, K. Pogany. *HIV nurse consultants:* C.J. Brouwer, D. Heida-Peters, E. Mulder, J.V. Smit, D. Struik-Kalkman. *HIV data collection:* T. van Niekerk. *HIV clinical virologists/chemists:* C. van Tienen.

**Maastricht UMC+, Maastricht:**
*HIV treating physicians:* S.H. Lowe*, A.M.L. Oude Lashof, D. Posthouwer, A. Stoop, M.E. van Wolfswinkel. *HIV nurse consultants:* R.P. Ackens, M. Elasri, K. Houben-Pintaric, J. Schippers. *HIV clinical virologists/chemists:* T.R.A. Havenith, M. van Loo.

**Frisius MC, Leeuwarden:**
*HIV treating physicians:* M.G.A. van Vonderen*, L.M. Kampschreur, S.E. van Roeden. *HIV nurse consultants:* M.C. van Broekhuizen, S. Faber *HIV clinical virologists/chemists:* A. Al Moujahid.

**Medisch Spectrum Twente, Enschede:**
*HIV treating physicians:* G.J. Kootstra*, C.E. Delsing. *HIV nurse consultants:* M. van der Burg-van de Plas, L. Scheiberlich.

**Noordwest Ziekenhuisgroep, Alkmaar:**
*HIV treating physicians:* W. Kortmann*, G. van Twillert*, R. Renckens, J. Wagenaar. *HIV nurse consultants & HIV data collection:* D. Ruiter-Pronk, B. Stander. *HIV clinical virologists/chemists:* J.W.T. Cohen Stuart, M. Hoogewerf, W. Rozemeijer, J.C. Sinnige.

**OLVG, Amsterdam:**
*HIV treating physicians:* K. Brinkman*, G.E.L. van den Berk, K.D. Lettinga, M. de Regt, W.E.M. Schouten, J.E. Stalenhoef, S.M.E. Vrouenraets. *HIV nurse consultants:* H. Blaauw, G.F. Geerders, M.J. Kleene, M. Knapen, M. Kok, I.B. van der Meché, A.J.M. Toonen, S. Wijnands, E. Wttewaal. *HIV clinical virologists:* D. Kwa, T.J.W. van de Laar.

**Radboudumc, Nijmegen:**
*HIV treating physicians:* R. van Crevel*, K. van Aerde, R.J.W. Arts, S.S.V. Henriet, H.J.M. ter Hofstede, J. Hoogerwerf, O. Richel, K. Stol. *HIV nurse consultants:* M. Albers, K.J.T. Grintjes-Huisman, M. de Haan, M. Marneef. *HIV clinical virologists/chemists:* M. McCall, J. Rahamat-Langendoen, E. Ruizendaal. *HIV clinical pharmacology consultant:* D. Burger.

**Rijnstate, Arnhem:**
*HIV treating physicians:* E.H. Gisolf*, M. Claassen, R.J. Hassing,. *HIV nurse consultants:* G. ter Beest, P.H.M. van Bentum, Y. Neijland, M. Valette. *HIV clinical virologists/chemists:* C.M.A. Swanink, M. Klein Velderman.

**Spaarne Gasthuis, Haarlem:**
*HIV treating physicians:* S.F.L. van Lelyveld*, R. Soetekouw. *HIV nurse consultants:* L.M.M. van der Prijt, J. van der Swaluw. *HIV clinical virologists/chemists:* J.S. Kalpoe, A. Wagemakers, A. Vahidnia.

**Medisch Centrum Jan van Goyen, Amsterdam:**
*HIV treating physicians:* F.N. Lauw, D.W.M. Verhagen. *HIV nurse consultants:* M. van Wijk.

**Universitair Medisch Centrum Groningen, Groningen:**
*HIV treating physicians:* W.F.W. Bierman*, M. Bakker, J. Kleinnijenhuis, E. Kloeze, A. Middel, D.F. Postma, Y. Stienstra, M. Wouthuyzen-Bakker. *HIV nurse consultants:* A. Boonstra, M.M.M. Maerman, D.A. de Weerd. *HIV clinical virologists/chemists:* M. Knoester, C.C. van Leer-Buter, H.G.M. Niesters, X.W. Zhou.

**Beatrix Kinderziekenhuis (Universitair Medisch Centrum Groningen), Groningen:**
*HIV treating physicians:* B.R. Brandsema, A.R. Verhage. *HIV nurse consultants:* N. van der Woude. *HIV clinical virologists/chemists:* M. Knoester, C.C. van Leer-Buter, H.G.M. Niesters, X.W. Zhou.

**Universitair Medisch Centrum Utrecht, Utrecht:**
*HIV treating physicians:* T. Mudrikova*, R.E. Barth, A.H.W. Bruns, P.M. Ellerbroek, M.P.M. Hensgens, J.J. Oosterheert, E.M. Schadd, A. Verbon, B.J. van Welzen. *HIV nurse consultants:* B.M.G. Griffioen-van Santen, L. van de Koolwijk, I. de Kroon. *HIV clinical virologists/chemists:* F.M. Verduyn Lunel, A.M.J. Wensing.

**Wilhelmina Kinderziekenhuis, UMC Utrecht, Utrecht:**
*HIV treating physicians:* Y.G.T. Loeffen, T.F.W. Wolfs. *HIV nurse consultants:* M. Kok. *HIV clinical virologists/chemists:* F.M. Verduyn Lunel, A.M.J. Wensing.

**Curaçao Medical Center, Willemstad (Curaçao):**
*HIV treating physicians:* E.O.W. Rooijakkers, D. van de Wetering. *HIV nurse consultants:* A. Alberto. *Data collection:* I. der Meer.

**Coordinating center**
*Board of directors:* M. van der Valk, S. Zaheri.
*HIV data analysis:* A.C. Boyd, D.O. Bezemer, V.W. Jongen, A.I. van Sighem, C. Smit, F.W.M.N. Wit.
*Data HIV data management and quality control:* M.M.J. Hillebregt, T.J. Woudstra, T. Rutkens
*HIV data monitoring:* D. Bergsma, J.M. Grolleman, L.E. Koster, K.J. Lelivelt, S.T. van Loenen,

M.J.C. Schoorl, K.M. Visser.
*HIV data collection:* K.J. Lelivelt, K.M. Visser, M. van den Akker, O.M. Akpomukai, R. Alexander, Y.M. Bakker, L. Bastos Sales, A. el Berkaoui, M. Bezemer-Goedhart, C.B.J. Bon, E.A. Djoechro, I. el Hammoud, M.R. Khouw, C.R.E. Lodewijk, E.G.A. Lucas, S. van Meerveld-Derks, M.A. van Montfoort, H.W. Mulder, L. Munjishvili, C.M.J. Ree, R. Regtop, A.F. van Rijk, Y.M.C. Ruijs-Tiggelman, P.P. Schnörr, R. van Veen, W.H.G. van Vliet-Klein Gunnewiek, E.C.M Witte.
*Patient registration:* D. Bergsma, Y.M.C. Ruijs-Tiggelman.
