# Supplementary material for: Labor Market Outcomes of People with HIV Pre- and Post-Diagnosis in the Netherlands
Source: Nat Commun. 2026 Jan 28;17:1110. doi: 10.1038/s41467-025-67799-x (PMC12855923; doi:10.1038/s41467-025-67799-x)
Supplement: Supplementary file 2 — Reporting Summary [file 41467_2025_67799_MOESM2_ESM.pdf]

nature portfolio

Corresponding author(s): Andrei Tuiu

Last updated by author(s): Nov 28, 2025

Reporting Summary

Nature Portfolio wishes to improve the reproducibility of the work that we publish. This form provides structure for consistency and transparency in reporting. For further information on Nature Portfolio policies, see our Editorial Policies and the Editorial Policy Checklist.

Statistics

For all statistical analyses, confirm that the following items are present in the figure legend, table legend, main text, or Methods section.

n/a	Confirmed	
☐	☒	The exact sample size (*n*) for each experimental group/condition, given as a discrete number and unit of measurement
☐	☒	A statement on whether measurements were taken from distinct samples or whether the same sample was measured repeatedly
☐	☒	The statistical test(s) used AND whether they are one- or two-sided<br>*Only common tests should be described solely by name; describe more complex techniques in the Methods section.*
☐	☒	A description of all covariates tested
☐	☒	A description of any assumptions or corrections, such as tests of normality and adjustment for multiple comparisons
☐	☒	A full description of the statistical parameters including central tendency (e.g. means) or other basic estimates (e.g. regression coefficient) AND variation (e.g. standard deviation) or associated estimates of uncertainty (e.g. confidence intervals)
☐	☒	For null hypothesis testing, the test statistic (e.g. *F*, *t*, *r*) with confidence intervals, effect sizes, degrees of freedom and *P* value noted<br>*Give P values as exact values whenever suitable.*
☒	☐	For Bayesian analysis, information on the choice of priors and Markov chain Monte Carlo settings
☒	☐	For hierarchical and complex designs, identification of the appropriate level for tests and full reporting of outcomes
☐	☒	Estimates of effect sizes (e.g. Cohen's *d*, Pearson's *r*), indicating how they were calculated

Our web collection on statistics for biologists contains articles on many of the points above.

Software and code

Policy information about availability of computer code

Data collection	No data collection was conducted for this study. Data sources are described below.
Data analysis	Data analysis is conducted using standard commands with the statistical software Stata, version 16, as well as the user-written command csdid for staggered difference-in-differences models.

For manuscripts utilizing custom algorithms or software that are central to the research but not yet described in published literature, software must be made available to editors and reviewers. We strongly encourage code deposition in a community repository (e.g. GitHub). See the Nature Portfolio guidelines for submitting code & software for further information.

Data

Policy information about availability of data

All manuscripts must include a data availability statement. This statement should provide the following information, where applicable:

- Accession codes, unique identifiers, or web links for publicly available datasets
- A description of any restrictions on data availability
- For clinical datasets or third party data, please ensure that the statement adheres to our policy

All results presented in the manuscript are calculated from non-public registry data from Centraal Bureau voor de Statistiek (CBS), accessed through the Remote Access environment. CBS was not involved in the calculation of any of the results presented. While the data are not publicly available, academic institutions can apply for access to the Remote Access environment through the CBS (for additional information, see https://www.cbs.nl/en-gb/our-services/customised-services-

microdata/microdata-conducting-your-own-research). ATHENA cohort data (without CBS data) used in this study are available upon reasonable request. Requests for data access can be made to: hiv.monitoring@amsterdamumc.nl. Requests will be reviewed on a case-by-case basis. Statistical information or data for separate research purposes from the ATHENA cohort can be requested by submitting a research proposal to SHM (https://www.hiv-monitoring.nl/english/research/research-projects/). The proposal will undergo review by representatives of SHM for evaluation of scientific value, relevance of the study, design, and feasibility, statistical power, and overlap with existing projects.

Research involving human participants, their data, or biological material

Policy information about studies with human participants or human data. See also policy information about sex, gender (identity/presentation), and sexual orientation and race, ethnicity and racism.

Reporting on sex and gender

In the manuscript, we refer to "registered gender" as it is reported by Statistics Netherlands based on the Personal Records Database (Dutch: BRP). It is defined as a binary variable taking the value of 0 for females and a 1 for males. In the analysis, we use the registered gender as a a matching variable and covariate. In the Heterogeneity analysis, we disaggregate the sample by registered gender and report descriptive results for each.

Reporting on race, ethnicity, or other socially relevant groupings

In the analysis, we report an individual's migration background, and, where applicable in the Heterogeneity analysis, the origin country of the individual themselves or their parents. Migration background is defined in three categories as: Native Dutch (the individual and both parents born in the Netherlands), 1st Generation Migrant (the individual is born outside of the Netherlands and at least one of their parents is born outside of the Netherlands) and 2nd Generation Migrant (the individual is born in the Netherlands and at least one parent is born outside of the Netherlands).

In the Heterogeneity analysis, we further disaggregate by the specific origin of the individual or their parents (where applicable), not accounting for generation. These categories are: Native Dutch; Middle East, North Africa (MENA) and Asia; Europe and North America; Suriname, Latin America and the Caribbean; Sub-Saharan Africa.

We do not use the these variables as proxies for any socially constructed variables, such as income or education.

Population characteristics

The population characteristics can be classified as demographic characteristics, labor market outcomes and HIV-specific characteristics. Demographic data are registered sex, birth year (based on which age at HIV diagnosis is constructed), migration background and highest level of education achieved. Labor market outcomes are employment for at least one month in a given year, work hours relative to the full-time equivalent (FTE), yearly income from work and disability insurance receipt for at least one month in a given year. HIV-specific characteristics are CD4 cell counts at the time of diagnosis, time in days from diagnosis to ART initiation, an indicator for being virally suppressed within 48 weeks of initiating ART and whether an individual has experienced an AIDS-defining event up to the time of diagnosis. We also classify people with HIV and their respective matched controls as having a non-late stage or late-stage HIV diagnosis based on the CD4 cell count, whether the person has a previous negative HIV test and whether they have had any AIDS-defining events.

Recruitment

People entering HIV care receive written material about participation in the ATHENA cohort, after which they are asked to consent verbally to the use of their routinely collected medical data for research and monitoring (i.e., an “opt-in” procedure).

We also use population registry data, for which no active recruitment was undertaken.

Ethics oversight

The data collected from participants in the ATHENA cohort is part of routine monitoring. At initiation, the cohort was approved by the institutional review board of all participating centers. The participants provide consent to use ATHENA data for research purposes and also allow linkage to other data sources as Statistics Netherlands. (https://www.hiv-monitoring.nl/en/what-we-do/information-people-living-hiv/patient-information-sheet). Information for participants about data linkage is listed on the website of Stichting HIV Monitoring (https://www.hiv-monitoring.nl/en/research-using-our-data/datakoppelingen).

Statistics Netherlands data is only accessible by authorized investigators. The usage and analysis of the combined dataset is allowed under the Dutch CBS (= Statistics Netherlands) law (https://wetten.overheid.nl/BWBR0015926/2022-03-02/#Hoofdstuk5_Paragraaf1_Artikel33). Output is independently checked by Statistics Netherlands.

Note that full information on the approval of the study protocol must also be provided in the manuscript.

Field-specific reporting

Please select the one below that is the best fit for your research. If you are not sure, read the appropriate sections before making your selection.

☐ Life sciences ☒ Behavioural & social sciences ☐ Ecological, evolutionary & environmental sciences

For a reference copy of the document with all sections, see nature.com/documents/nr-reporting-summary-flat.pdf

Behavioural & social sciences study design

All studies must disclose on these points even when the disclosure is negative.

Study description

Quantitative study of the causal effects of receiving an HIV diagnosis on individuals' labor market outcomes.

Research sample

The main sample consists of 5,960 individuals with HIV diagnosed between 2010 and 2022. Each individual is matched to 10 random

Research sample	individuals from the general population with the same birth year, registered sex, migration background and highest level of education achieved. Matching is conducted with replacement, meaning that an individual in the control group might be duplicated if they are matched to several people with HIV with the same demographic characteristics. This leads to a control group of 59,600 individuals. Matching is conducted in order to ensure that the control group is comparable in observable characteristics with the selected sample of people with HIV. The sample is not representative of the general Dutch population.<br><br>Among people with HIV, the median age in the year before diagnosis is 42, 87.6% of individuals are male, 36.3% have a low level of education (pre-vocational secondary education and/or the first three years of senior general secondary education or pre-university education), 29.0% have a medium level of education (completed senior general secondary education or pre-university education), 13.5% have a high level of education (completed higher vocational education or university).66.4% of individuals are born in the Netherlands, with both parents born in the Netherlands, 23.9% are born abroad with at least one parent born abroad, and 9.7% are born in the Netherlands, with at least one parent born abroad. The same statistics are true among the matched controls, due to the matching procedure.
Sampling strategy	All individuals initiating HIV care in the Netherlands are asked to participate in the ATHENA cohort.<br>Statistics Netherlands data covers the entire population of individuals registered in the Netherlands.<br>Further restrictions are applied in order to create the final study sample, as described below.
Data collection	Data is collected by Statistics Netherlands (CBS) via administrative registries, and by Stichting HIV Monitoring (SHM) via the ATHENA cohort. At enrollment into the ATHENA cohort, the following demographic information was collected: year of birth, country of birth, sex assigned at birth, gender identity (if different from sex at birth), and most likely transmission route of HIV. Data from routine visits are extracted from patient records by trained data collectors or automatically (lab based data).
Timing	The data used cover the period 2003 - 2022.
Data exclusions	People with HIV: we restrict the universe of 28,294 people with HIV whose clinical data could be linked to the Statistics Netherlands records to individuals diagnosed between the ages of 28 and 62 (n = 21,117). Next, we restrict the data to individuals diagnosed in the Netherlands (n = 18,165). We further restrict to individuals diagnosed between 2010 and 2022 (n = 7,113).<br><br>Finally, we further restrict the sample to individuals who are observed for seven consecutive years prior to diagnosis in the employment and welfare data and who have at most two years of missing income data (n = 5,960). Out of the 356 individuals for whom income data is missing, we are able to impute incomes for 252 The purpose of these restrictions is to ensure that the outcomes of individuals with HIV can be continuously observed in the pre-diagnosis period.<br><br>Matched controls: We exclude from the universe of individuals ever registered in the Netherlands since 1995 individuals who are not observed in the employment, welfare, and income datasets for at least 7 consecutive years. The remaining 17 million people form the pool eligible match donors. The group of matched controls is then randomly selected in a process of 10-to-1 exact matching with replacement..<br><br>In the descriptive and difference-in-differences analysis for employment and disability insurance, all individuals in the sample are included. For the income analysis, we exclude 104 individuals with missing income data for whom income could not be imputed, as well as their matched controls. For the work hours analysis, we exclude 326 individuals whose work hours are not observed for at least four years before diagnosis, as well as the individuals they are matched to.
Non-participation	According to the 2024 HIV Monitoring Report published by Stichting HIV Monitoring, of the 35,017 people with HIV ever registered by SHM, 973 did not give permission for data collection. People who retract their informed consent are immediately removed from ATHENA databases.<br><br>The data collected by Statistics Netherlands is based on administrative records, thus non-participation is not a concern.
Randomization	Allocation into receiving an HIV diagnosis is not random. It is controlled for through the matching procedure, which selects a control group similar to the group of people with HIV. In addition, the staggered difference-in-differences methodology applied allows us to control for unobserved individual- and time-specific characteristics.

Reporting for specific materials, systems and methods

We require information from authors about some types of materials, experimental systems and methods used in many studies. Here, indicate whether each material, system or method listed is relevant to your study. If you are not sure if a list item applies to your research, read the appropriate section before selecting a response.

Materials & experimental systems

n/a	Involved in the study	
☒	☐	Antibodies
☒	☐	Eukaryotic cell lines
☒	☐	Palaeontology and archaeology
☒	☐	Animals and other organisms
☐	☒	Clinical data
☒	☐	Dual use research of concern
☒	☐	Plants

Methods

n/a	Involved in the study	
☒	☐	ChIP-seq
☒	☐	Flow cytometry
☒	☐	MRI-based neuroimaging

Clinical data

Policy information about clinical studies

All manuscripts should comply with the ICMJE guidelines for publication of clinical research and a completed CONSORT checklist must be included with all submissions.

Clinical trial registration	N/A
Study protocol	The ATHENA cohort profile can be found in: Boender, T. S. et al. AIDS Therapy Evaluation in the Netherlands (ATHENA) national observational HIV cohort: cohort profile. BMJ Open 8, e022516 (2018). https://doi.org/10.1136/bmjopen-2018-022516
Data collection	Data collection for ATHENA started in 1998 and is on-going. Data are collected at the centers where individuals are in HIV care.
Outcomes	Clinical outcomes are not used in the study.

Plants

Seed stocks	N/A
Novel plant genotypes	N/A
Authentication	N/A
